# Financial Instability and Delays in Access to Sexual and Reproductive Health Care Due to COVID-19

**DOI:** 10.1089/jwh.2021.0493

**Published:** 2022-04-19

**Authors:** Megan L. Kavanaugh, Zoe H. Pleasure, Emma Pliskin, Mia Zolna, Katrina MacFarlane

**Affiliations:** ^1^ Research Division, Guttmacher Institute, New York, New York, USA.; ^2^ Research Division, Formerly of the Guttmacher Institute, New York, New York, USA.

**Keywords:** COVID-19, contraception, financial instability, health care access

## Abstract

**Objective:**

To identify prevalence of, and patient and clinic characteristics associated with, delays in access to sexual and reproductive health (SRH) care due to the COVID-19 pandemic across three states with varying COVID-19 context and state government response.

**Methods:**

We weighted data collected between May 2020 and May 2021 from monthly and biannual follow-up surveys of patients seeking family planning care at a publicly supported health center in Arizona (*N* = 538), Iowa (*N* = 341), and Wisconsin (*N* = 568), who reported on experiences 6–18 months before the survey. We conducted multivariable logistic regression analyses to identify characteristics associated with delays in accessing SRH care due to COVID-19, with specific attention to associations between patients' financial instability and experiencing delays.

**Results:**

Between May 2020 and May 2021, over half of respondents in Arizona (57%), 38% in Iowa, and 30% in Wisconsin indicated that they were either unable to access or delayed accessing SRH care or a contraceptive method due to the COVID-19 pandemic. In Arizona and Wisconsin, in multivariable models, respondents who had experienced financial instability due to being out of work, having fallen behind on key life payments, or because of a job reduction or loss due to COVID-19 had increased odds of experiencing COVID-19-related SRH care delays (Arizona adjusted odds ratio [aOR] = 2.6, *p* = 0.01 and Wisconsin aOR = 6.0, *p* < 0.001).

**Conclusions:**

Access to contraception was curtailed during the COVID-19 pandemic, especially for those who experienced employment and financial instability. Individuals' and clinics' ability to mitigate these effects were likely dependent on state context and response to the pandemic, among other factors.

## Introduction

The COVID-19 pandemic in the United States suspended all areas of individuals' lives, including the economic, health, and social spheres, with far-reaching impacts in each. These broad impacts quickly influenced peoples' ideas about starting or growing a family: two separate national studies conducted in early May 2020 documented individuals' changing pregnancy desires in the context of COVID-19.^1,2^ In the first study, two-thirds of US adults considered it a bad time for individuals and couples to try to get pregnant^1^ and in the second, one-third of cisgender US women wanted to get pregnant later or wanted fewer children because of the pandemic.^2^

A third national study drawing from social media at the same time highlighted that drops in desire to be pregnant were more common among those who simultaneously reported being unable to afford food, transportation, and/or housing.^3^

Findings regarding individuals' desires to delay and/or prevent pregnancy during the pandemic underscore a potentially increased need for contraception to achieve these goals. However, while the pandemic has affected individuals' desires regarding pregnancy, so too has it impacted how sexual and reproductive health (SRH) care are provided. SRH care providers reported a range of disruptions in how they were able to provide contraception to their patients, including postponing intrauterine device insertions or Depo-Provera^®^ appointments,^4,5^ shifting staff away from SRH care toward COVID-related care, and reducing clinic hours or stopping in-person SRH care altogether.^6^

These disruptions in care coincided with national conversations early in the pandemic as to whether contraceptive care, and SRH care more broadly, should be deemed essential when considering where to shift health care resources during a pandemic emergency; many professional and medical societies came down firmly in support of the essential nature of contraception to individuals' health and autonomy.^7,8^ Notably, some providers of SRH care nimbly pivoted in how they provided contraceptive care and methods to their patients, including changes such as establishing virtual/telehealth alternatives for appointments or getting methods to patients in innovative ways such as by mail or parking lot pickups.^4,6,9,10^

However, patients themselves reported concerns about seeking non-COVID-related health care (68% of US adults)^1^ and experiencing delays and/or cancellations in getting their contraceptive care or method in the first few months of the pandemic (33% of cisgender women).^2^ These delays in care were more common among individuals who reported being more financially worse off compared to a year prior (42% vs. 29% among those not worse off), who also more commonly reported worrying about being able to take care of their children due to COVID-19.^2^ Another national study of US adults later in the pandemic (November–December 2020) documented that younger people (ages 18–34) more commonly reported delays or inability in getting their birth control method due to COVID-19 compared to those older than 35 years.^11^

Potential impacts of delaying access to broad SRH care for individuals include pregnancies, complications from undetected or untreated sexually transmitted infections (STIs) such as infertility, pelvic inflammatory disease, and ectopic pregnancies, and other gynecologic morbidities^12^; thus, determining the extent to which individuals experience these delays is critical.

While data at the national level regarding impacts of the COVID-19 pandemic and its fallout on people's SRH help to provide an overall picture of what is happening in the United States, they may also mask important variation at the state level due to state-specific context regarding the pandemic and response. In addition, data on delays to accessing SRH care from 2020 may overstate the extent to which clinic-level closures or reductions in SRH services in the early months of the pandemic may have led to patient-level delays. Our study builds on previous national-level evidence of delays in access to SRH care experienced early in the COVID-19 pandemic by examining the extent to which individuals experienced delays over the course of a year into the pandemic in three states, each with a different COVID-19 epidemiological context and governmental response.

All three states faced rapid surges in COVID-19 infections at various points throughout 2020: Arizona was a hot spot in the summer of 2020 and again in the winter of 2020–2021, reporting the worst COVID-19 infection rate of anywhere in the world by the new year,^13^ Iowa was reporting the highest positive coronavirus cases in the United States by August 2020,^14^ and Wisconsin also reported huge surges by November 2020.^15^ In a 2020 Berkeley report ranking COVID-19 responses based on three key metrics (testing, infections, and deaths), Wisconsin was ranked 32nd, Arizona was 47^th^, and Iowa was 49th among the states for their COVID-19 response.^16^

Our study capitalized on ongoing longitudinal data collection activities with family planning patients in Arizona, Iowa, and Wisconsin under a broader study, the Reproductive Health Impact Study, which examines the impact of state and federal policy changes on patients accessing publicly supported family planning care in these states. Drawing on data collected between May 2020 and May 2021, this study documents the prevalence of patient reports of delays in access to SRH care due to the COVID-19 pandemic in each of these three states, with specific attention to associations between patients' financial instability and experiencing delays.

## Methods

### Data collection

Data for this analysis come from the Reproductive Health Impact Study, a longitudinal study of individuals seeking care at publicly supported family planning sites in Iowa (April 2018–February 2019), Arizona (May 2019–January 2020), and Wisconsin (February 2020–June 2021). Patients were eligible to participate in the study if they reported they were assigned female on their birth certificate, were 15 years of age or older, sought family planning care* at an eligible site during its fielding period, and did not have a confirmed pregnancy at the time of their appointment.

Surveys were available in both English and Spanish. Eligible sites in Iowa served 100 or more family planning patients annually and, in Arizona and Wisconsin to shorten fielding periods at each site, eligible sites served 200 or more patients annually, based on information from the Guttmacher Institute's most recent census of all known publicly supported family planning sites in the United States^17^ and supplemented by health center administrators' reports of annual patient caseload at the time of site recruitment. Across all three states, 58 sites participated.

Survey respondents who completed the self-administered baseline survey at the health center site were offered the opportunity to receive monthly (participants older than 18 years) and biannual surveys (all participants) through short message service texts and/or emailed survey links for 2 years starting 1 month after completing the baseline survey. A total of 2928 participants consented to receive longitudinal surveys across the states: 633 of 1424 respondents in Iowa (44%), 1152 of 1990 in Arizona (58%), and 1142 of 1676 in Wisconsin (68%). Respondents were provided small monetary incentives for participating in the study, with differing amounts across states and survey waves.

Monthly and biannual follow-up surveys were still ongoing, but at different stages in each study state when the COVID-19 pandemic resulted in widespread community and societal shutdowns in many parts of the country in Spring 2020. At this time in Iowa, respondents were ∼12–18 months post-baseline, whereas Arizona respondents were about 6–12 months post-baseline and some Wisconsin respondents were just starting their monthly follow-up surveys. In addition to existing survey content, which focused on SRH care access and barriers, contraceptive use and preferences, pregnancy attitudes, and life context, we added four additional survey items to assess the impact of the COVID-19 pandemic on respondents' lives, including access to SRH care, to all monthly and biannual surveys across all three study states starting April 1, 2020.

The research study protocol and addenda for the Reproductive Health Impact Study were approved by our and our data management partner's respective institutional review boards.

### Analytic sample

Our analysis is limited to data from respondents who answered at least one of the four COVID-19 questions on either a monthly or biannual survey between May 2020 and May 2021 and completed the fourth biannual survey in Iowa (24 months after baseline, between April 2020 and February 2021), the second biannual survey in Arizona (12 months after baseline, between May 2020 and January 2021), or the first biannual survey in Wisconsin (6 months after baseline, between August 2020 and May 2021).

For respondents who had not yet received their first biannual survey by May 2021 (Wisconsin only, given its later stage of fielding: *N* = 369, 25.5% of total sample), we drew upon data from their last completed monthly survey. We chose to limit the analysis to these survey rounds since they occurred during key peaks of the COVID-19 pandemic and relied on data from biannual surveys when possible because these included more survey content.

Analytic sample data were weighted to account for the sampling design using a multistep process. In each state, respondents were weighted up to their site's total patient caseload and then up to the total patient caseload served within their site's stratum (based on site type and caseload size). We further adjusted the weights to account for the total number of patients served in strata where we had no data representation in our analytic sample. The universe control data came from the same set of data used to identify site eligibility into the sample, a 2018 Guttmacher census of publicly supported sites supplemented by administrator reports (Reproductive Health Impact Study 2018 Family Planning Clinic Census, unpublished tabulations).

Our analytic sample did not include respondents from Federally Qualified Health Centers of any size in any of the three states or hospitals of any size in Arizona, either because sites of these types did not participate in our study or because respondents from sites with excessively large sampling variance were removed from our analytic sample; therefore, there were no “like” respondent data that would have adequately represented the universe of patients seen at these sites and so we adjusted the universe control data to exclude patient counts from these center types accordingly for each state. Finally, we trimmed the weights for Iowa and Wisconsin to minimize skewness and spread, resulting in a coefficient of variation of the weights below 0.8 for each state.

### Analysis

We examined respondent- and site-level characteristics as independent variables associated with our outcome of interest focused on experiences of COVID-19-related delays in access to SRH care. Respondents' age, race and ethnicity,^†^ income as a percentage of federal poverty level, educational attainment, and the language in which the survey was taken come from baseline surveys, as none of these items was asked in subsequent surveys.

We used respondents' contraceptive method use at baseline rather than data from any subsequent time point for this metric, as we conceived of respondents' preliminary contraceptive use as potentially being malleable to delays in accessing SRH care due to COVID-19 at subsequent time points. Given our focus on accessing care related to contraception, we classified respondents' method use by whether a provider is required to provide, prescribe, or remove the contraceptive method (provider involved) versus not. We classified respondents who indicated using permanent methods (female sterilization or vasectomy) at baseline as being a nonprovider-involved method user because these methods likely would have required much less contact with a provider during the timeframe of follow-up of this study than other methods.

We categorized where respondents sought care at baseline according to whether the site received Title X funding—a federal grant program aimed at providing comprehensive family planning and related preventive health care on a sliding fee scale—at the time of baseline survey fielding to identify who may have obtained care at reduced or no cost.^18^ Self-reported sexual orientation, current health insurance status, and employment status were collected in both baseline and biannual surveys, and we drew these metrics from the latest time point collected for each participant, given both their documented fluidity in general,^19,20^ as well as our desire to capture a more recent COVID-related change.

We created a composite measure of financial instability, which combined responses to an item that asked respondents whether they fell behind on their rent, mortgage, or student loans in the past month or 6 months with responses to having had a personal or close family/friend experience with job-related reductions or losses due to COVID-19. The former item was included in all monthly and biannual surveys, while the latter was added following the onset of the pandemic; we focused on the latest response for biannual respondents and any response to the item from months one through five for monthly respondents for this analysis. Any affirmative response to either item was recoded to a “yes” on our financial instability composite variable.

Finally, we also included an item that asked respondents about their own or a close family/friend's experience with presumed or confirmed contraction of COVID-19 as an independent variable.

Our outcome of interest drew on two items from the monthly and biannual surveys specific to understanding respondents' broad SRH care-related experiences in the context of the COVID-19 pandemic: (1) whether they had to delay, cancel, or skip SRH care and (2) whether they were unable to get, or delayed in getting, their birth control method due to COVID-19. We consolidated these two items into a composite measure of COVID-19 delays in contraceptive care, coded as “yes” for any affirmative response to either of these items over the May 2020–May 2021 data collection period.

We first examined the distribution of individuals in our analytic sample by key sociodemographic and SRH characteristics within each state. Next, we examined these sociodemographic and SRH characteristics by our key outcome of interest focused on COVID-19-related delays to SRH care by state. Finally, for each state, we used multivariable logistic regression to estimate adjusted odds ratios (aORs) for the relationship between respondent characteristics and our composite measure of COVID-19-related delays on SRH care as the outcome variable. All statistical analyses were performed using the “svy” prefix command within Stata version 16.1^21^ to account for the study's complex sample design.

## Results

### Sample characteristics

Table 1 displays sociodemographic and SRH characteristics of survey respondents in Arizona (*N* = 538), Iowa (*N* = 341), and Wisconsin (*N* = 568). In all states, over half were between 20 and 29 years of age. In Arizona, the largest share of respondents was Hispanic (46%), while in Iowa and Wisconsin, the majority of respondents was non-Hispanic white (74% and 41%, respectively). In each state, at least 20% of respondents identified as non-straight (lesbian, gay, bisexual, queer, pansexual, or other), and about one-third were below 100% of the federal poverty level. Less than 5% of the sample in each state took the survey in Spanish. Private health insurance was the most common coverage reported in Arizona (48%) and Iowa (65%), while public insurance was the most common in Wisconsin (57%). Most respondents across states (84%–90%) reported being employed or being a student.

**Table 1.** Weighted Distributions of Selected Sociodemographic Characteristics Among Analytic Sample by State, 2020–2021

	Arizona		Iowa		Wisconsin	
	*N*	Weighted %	*N*	Weighted %	*N*	Weighted %
Total	538	100.00	341	100.00	568	100.00
Age (years)^a^						
15–19	74	14.50	50	14.84	82	14.59
20–24	207	39.35	133	34.59	168	26.76
25–29	122	21.00	77	21.34	139	26.63
30–34	70	13.43	39	12.33	89	17.62
35+	64	11.72	42	16.90	90	14.40
Race/ethnicity^a^						
White non-Hispanic	234	40.91	245	74.43	333	41.09
Black non-Hispanic	31	5.53	26	8.92	112	33.62
Other non-Hispanic	43	7.37	26	6.36	34	5.70
Hispanic	229	46.19	41	10.29	88	19.59
Sexual orientation^b^						
Straight	401	77.26	261	79.87	417	73.81
Lesbian, gay, bisexual, queer, pansexual, or other	124	22.74	77	20.13	140	26.19
Income level^a^						
<100% FPL	144	35.75	96	31.12	152	33.97
100%–199% FPL	138	26.84	86	27.44	180	36.85
200%+ FPL	175	37.41	110	41.43	160	29.18
Education^a^						
HS graduate, GED, or less	152	29.34	77	25.37	219	43.17
Some college or associate degree	228	43.81	175	47.51	227	37.55
College graduate or more	157	26.84	89	27.12	120	19.28
Survey language^a^						
English	519	95.57	340	99.74	554	96.89
Spanish	19	4.43	1	0.26	14	3.11
Current health insurance^b^						
Private insurance	247	47.50	215	64.49	212	35.93
Public insurance	141	29.22	99	29.28	275	56.59
No insurance	115	23.28	22	6.23	36	7.49
Employment^b^						
Employed or student	435	83.65	292	89.47	484	87.45
Not employed nor student	80	16.35	37	10.53	61	12.55
Experienced financial hardship^c^	459	82.01	257	77.91	341	62.77
Current method use^a^						
Provider involved	337	67.57	249	72.77	411	66.66
Nonprovider involved or permanent	112	18.09	49	14.69	99	19.61
No method	83	14.34	33	12.54	55	13.74
Received care at a Title X funded site at baseline^a^	352	74.27	267	84.97	242	24.34
Self, partner, or family member diagnosed with COVID-19	364	67.49	128	35.67	205	37.41

*Notes:* Respondents' method use is considered to be “provider involved” if a provider is required to provide, prescribe, or remove the contraceptive method (intrauterine device, implant, shot, ring, pill, or patch). Baseline use of a permanent method (tubal ligation or partner vasectomy) is considered nonprovider involved because these methods likely would have required much less contact with a provider during the timeframe of follow-up of this study than would other methods. Respondents were eligible if they were not pregnant at baseline, were assigned female at birth, and were at a clinic for a family planning service, including birth control counseling, a birth control method, an STI test or treatment, HPV vaccination, a pregnancy test, or an annual GYN appointment. Respondents also had to have completed one biannual survey during May 2020–May 2021 or a monthly survey if they had not received a biannual during this time frame. Public insurance options include Medicaid, Medicare, Tricare, Indian Health Service, and SFPP. Private insurance includes employer-based plans and plans purchased on the marketplace or exchange. Other insurance includes those who selected “other” as their insurance coverage, and whose write-in responses could not be recoded as another type of insurance.

^a^ Question asked at baseline.

^b^ For Wisconsin respondents who had only answered monthly and not biannual surveys, baseline answers to these variables are used.

^c^ Composite measure created from variables that measure falling behind on rent, mortgage, or student loans in the past month or 6 months or having had a personal or close family/friend experience job-related reductions or losses due to COVID-19.

FPL, federal poverty level; GED, General Educational Development; GYN, gynecological; HPV, human papillomavirus; HS, high school; SFPP, State Food Purchase Program; STI, sexually transmitted infection.

In all states, most respondents (63%–82%) indicated experiencing financial instability—either by having fallen behind on their rent, mortgage, or student loans^‡^ or in terms of a COVID-19-related job loss or reduction. Across states, the majority of respondents (86%–87%) was contraceptive users, with most of this use being provider-involved methods (67%–73%). Most respondents in Iowa (85%), about three quarters of respondents in Arizona (74%), and only a quarter of respondents in Wisconsin (24%) received care at baseline at a health care center that received Title X grant support to provide family planning services. Arizona respondents reported the highest levels of having a personal or close COVID-19 diagnosis (68%) compared to respondents in both Wisconsin (37%) and Iowa (36%).

### Characteristics associated with delays in SRH care due to COVID-19

In Arizona, over half of respondents indicated experiencing delays in accessing SRH care due to COVID-19 (57%), while 38% of respondents in Iowa and 30% in Wisconsin reported this outcome (Table 2). After adjusting for respondent demographic and sexual and reproductive characteristics, there were few significant differences in experiencing this outcome by population group across these three states. One exception to this finding was in Wisconsin, where individuals with incomes at 200% or more of the federal poverty level at baseline had 1.6 times higher odds of experiencing delays in SRH care due to COVID-19 compared to those with incomes <100% of the federal poverty level (*p* = 0.05).

**Table 2.** Adjusted Percentages and Odds Ratios from Multivariable Logistic Regression Analyses Assessing Associations Between Selected Sociodemographic Characteristics and Delays in Access to Sexual and Reproductive Health Care Due to COVID-19 in Arizona, Iowa, and Wisconsin, 2020–2021

	Arizona (*N* = 406)			Iowa (*N* = 270)			Wisconsin (*N* = 439)		
	Weighted %	aOR (CIs)	*p*	Weighted %	aOR (CIs)	*p*	Weighted %	aOR (CIs)	*p*
Total	56.62			38.26			29.79		
Age (years)^a^									
15–19	54.79	1.00		38.17	1.00		40.88	1.00	
20–24	60.79	1.76 (0.45–6.85)	0.36	38.73	1.22 (0.24–6.13)	0.79	26.04	0.50 (0.19–1.32)	0.14
25–29	53.60	1.57 (0.49–5.02)	0.39	42.47	1.97 (0.30–13.12)	0.43	32.20	0.88 (0.44–1.75)	0.68
30–34	60.88	2.41 (0.39–14.90)	0.29	32.15	0.74 (0.11–4.97)	0.73	23.28	0.34 (0.07–1.68)	0.16
35+	46.28	0.82 (0.22–3.12)	0.74	36.50	0.87 (0.10–7.35)	0.89	28.95	1.03 (0.45–2.34)	0.95
Race/ethnicity^a^									
White non-Hispanic	58.57	1.00		34.57	1.00		19.96	1.00	
Black non-Hispanic	61.28	1.33 (0.55–3.25)	0.47	59.23	1.31 (0.54–3.18)	0.50	41.30	2.36 (0.57–9.81)	0.21
Other non-Hispanic	45.74	0.70 (0.31–1.61)	0.35	37.74	1.41 (0.51–3.89)	0.46	31.08	0.69 (0.15–3.19)	0.60
Hispanic	56.33	1.08 (0.63–1.82)	0.76	49.52	2.65 (0.51–13.69)	0.21	30.36	1.64 (0.22–12.09)	0.60
Sexual orientation^b^									
Straight	53.75	1.00		34.52	1.00		28.72	1.00	
Lesbian, gay, bisexual, queer, pansexual or other	66.05	1.89 (0.98–3.64)	0.06	54.03	2.53 (1.06–6.04)	**0.04**	31.82	1.41 (0.84–2.34)	0.17
Income level^a^									
<100% FPL	55.36	1.00		47.26	1.00		28.06	1.00	
100%–199% FPL	60.48	1.18 (0.46–3.02)	0.69	37.27	0.56 (0.30–1.05)	0.07	29.83	1.48 (0.68–3.24)	0.29
200%+ FPL	58.82	1.27 (0.59–2.76)	0.49	32.33	0.41 (0.13–1.32)	0.12	28.46	1.62 (1.01–2.61)	**0.05**
Education^a^									
HS graduate, GED, or less	51.89	1.00		46.81	1.00		33.21	1.00	
Some college or associate degree	61.50	1.17 (0.77–1.76)	0.41	39.26	0.87 (0.22–3.45)	0.82	26.57	1.15 (0.58–2.26)	0.67
College graduate or more	54.16	0.79 (0.34–1.84)	0.53	28.49	0.70 (0.17–2.98)	0.59	29.14	1.63 (0.55–4.88)	0.35
Current health insurance^b^									
Private insurance	58.88	1.00		38.05	1.00		23.84	1.00	
Public insurance	55.57	1.16 (0.51–2.59)	0.69	35.93	0.92 (0.18–4.59)	0.91	31.18	1.66 (0.91–3.04)	0.09
No insurance	49.64	0.77 (0.34–1.76)	0.48	57.58	2.45 (0.40–15.15)	0.29	25.19	0.69 (0.33–1.45)	0.30
Employment^b^									
Employed or student	58.79	1.00		36.06	1.00		28.77	1.00	
Not employed nor student	48.76	0.79 (0.25–2.53)	0.65	42.86	0.73 (0.16–3.35)	0.65	34.97	1.19 (0.21–6.84)	0.83
Experienced financial hardship^c^									
No	33.23	1.00		22.43	1.00		6.69	1.00	
Yes	61.83	2.60 (1.29–5.21)	**0.01**	42.74	2.59 (0.54–12.44)	0.20	43.85	6.03 (2.49–14.61)	**<0.001**
Current method use^a^									
Provider involved	57.61	1.00		36.29	1.00		26.94	1.00	
Nonprovider involved or permanent	52.84	0.77 (0.39–1.53)	0.40	47.35	1.28 (0.55–2.98)	0.52	38.87	0.88 (0.12–6.22)	0.89
No method	56.48	1.01 (0.39–2.61)	0.98	32.29	0.72 (0.23–2.20)	0.51	31.25	1.72 (0.18–16.23)	0.61
Received care at a Title X funded site at baseline^a^									
No	63.65	1.00		42.67	1.00		32.67	1.00	
Yes	54.18	0.66 (0.33–1.31)	0.19	37.47	0.78 (0.16–3.93)	0.73	20.76	0.88 (0.17–4.63)	0.87
Self, partner, or family member diagnosed with COVID-19									
No	45.41	1.00		29.83	1.00		18.41	1.00	
Yes	62.02	1.62 (0.77–3.37)	0.17	53.46	1.76 (1.05–2.97)	**0.04**	48.78	2.56 (1.09–6.00)	**0.03**

Associations between respondent characteristics and the outcome that were significant at *p* ≤ 0.05 are represented in bold font.

Only respondents with nonmissing answers to the demographic questions were included in the adjusted models. *Notes:* Respondents' method use is considered to be “provider involved” if a provider is required to provide, prescribe, or remove the contraceptive method (intrauterine device, implant, shot, ring, pill, or patch). Baseline use of a permanent method (tubal ligation or partner vasectomy) is considered nonprovider involved because these methods likely would have required much less contact with a provider during the timeframe of follow-up of this study than would other methods. Respondents were eligible if they were not pregnant at baseline, were assigned female at birth, and were at a clinic for a family planning service, including birth control counseling, a birth control method, an STI test or treatment, HPV vaccination, a pregnancy test, or an annual GYN appointment. Respondents also had to have completed one biannual survey during May 2020–May 2021 or a monthly survey if they had not received a biannual during this time frame. Public insurance options include Medicaid, Medicare, Tricare, Indian Health Service, and SFPP. Private insurance includes employer-based plans and plans purchased on the marketplace or exchange. Other insurance includes those who selected “other” as their insurance coverage, and whose write-in responses could not be recoded as another type of insurance.

^a^ Question asked at baseline.

^b^ For Wisconsin respondents who had only answered monthly and not biannual surveys, baseline answers to these variables are used.

^c^ Composite measure created from variables that measure falling behind on rent, mortgage, or student loans in the past month or 6 months or having had a personal or close family/friend experience job-related reductions or losses due to COVID-19.

aOR, adjusted odds ratio; CI, confidence intervals.

In addition, Iowan respondents who were not straight—that is, they identified as lesbian, gay, bisexual, queer, pansexual, or something else—had greater odds of experiencing COVID-19-related delays in accessing SRH care than those identifying as straight (*p* = 0.04). Finally, respondents in Iowa and Wisconsin who had experienced a COVID-19 diagnosis either personally or as a close partner or family member had higher odds of experiencing COVID-19 SRH access delays than those who had not (adjusted odds ratio [aOR] = 1.8, *p* = 0.04 in Iowa and aOR = 2.6, *p* = 0.03 in Wisconsin).

In Arizona and Wisconsin, respondents' financial instability—determined either through having fallen behind on key life payments or by indicating a job reduction or loss due to COVID-19—was associated with increased odds of experiencing COVID-19-related SRH care delays. In Arizona, those who experienced financial disruption had over two and a half times higher odds of experiencing these delays than those without the same financial strains (aOR = 2.6, *p* = 0.01), while Wisconsin respondents with these financial strains had six times higher odds of this outcome (aOR = 6.0, *p* < 0.001) than their counterparts. Across all three states, neither insurance coverage nor having received SRH care at a Title X funded site at baseline was significantly associated with experiencing delays in accessing SRH care due to COVID-19.

## Discussion

Access to contraception and broader SRH care was curtailed during the COVID-19 pandemic, and for residents of Arizona, Iowa, and Wisconsin, who were experiencing concurrent employment and financial instability, these delays were heightened. The majority of Arizonan family planning patients experienced delays in accessing SRH care due to COVID-19, with Iowans and Wisconsinites experiencing similar and less frequent experiences of these delays (about 30%–40%) than Arizonans. These differing levels of impact may reflect the varying levels of COVID-19 community spread^22^ and varying government responses for disease mitigation across these state contexts, as well as the different time periods of the pandemic during which respondents from each state participated in the study.

In Arizona and Wisconsin, COVID-19-related SRH access impacts were disproportionately shouldered by individuals experiencing financial instability, who likely have fewer resources and financial supports to mitigate the economic fallout of the pandemic. Not surprisingly, these individuals are the same ones who already were experiencing greater structural barriers to SRH care than their wealthier, more resourced counterparts, even before the pandemic.

Our state-specific findings revealing experiences over a full year of the pandemic align with national-level evidence from early on regarding the heightened delays to SRH care experienced among individuals with financial instability,^2^ the very people whom the federally funded family planning program Title X was designed to serve. The lack of significant findings for this same relationship in Iowa may be due to a smaller sample size in this state compared to the other two states and/or differential demographics of respondents across the states, among other potential factors.

Our findings covering a year of the pandemic indicate that evidence of access barriers early in the pandemic was a harbinger of more sustained contraceptive access issues for patients, especially for those with the fewest resources to navigate these barriers. Particularly for users of long- and short-acting hormonal methods of contraception that commonly require a visit to a health care provider, which represent between 1/4 and 1/3 of all method use in these three states,^23^ long-term access delays to obtaining and/or refilling one's method can seriously inhibit individuals' ability to prevent pregnancy over a year.

In addition, given that the SRH care visit is the primary source of all medical care for over 2/3 of individuals seeking this care at publicly supported health facilities, delays in access to that care can have broader consequences for individuals' overall physical and mental health.^24^

Given the link between employment and health insurance coverage and the projections of job loss associated with the COVID-19 pandemic,^25^ it stands to reason that having and maintaining health insurance coverage would buffer some of the access barriers differentially experienced among individuals experiencing financial instability. However, our findings revealed no association between having and type of health insurance coverage and COVID-19-related access delays, suggesting at least two possible explanations. First, our sample was initially recruited at health care sites that received public support for family planning service delivery—in particular Title X funding—which provides low- to no cost care to low- or no income individuals.

One's own health insurance coverage plays less of a role in mitigating financial barriers when care is already subsidized. Our finding highlighting increased delays in SRH care for individuals in Wisconsin with higher incomes than the very lowest income levels may hint at unique access challenges experienced by those who are just over the eligibility threshold for no cost care, but not able to afford even the low cost that they are assigned on a sliding fee scale. Second, the homogeneity of experiencing COVID-19-related delays to care across most demographic characteristics within our sample—few differences in this experience by age, income level, and education, for example—suggests that access barriers may be less directly related to cost and reveals the ubiquity of this experience and the minimal influence that individual-level factors have to mitigate these impacts.

Beyond financial instability, there were few differences across respondents in each state with regard to experiencing COVID-19-related delays in SRH care, suggesting that these impacts were widespread across state-specific populations who may be in need of publicly supported family planning care. Notably, in Iowa, a state ranked 18th for being lesbian, gay, bisexual, transgender, queer/questioning (LGBTQ) friendly,^26–28^ and where COVID-related job losses were higher for non-straight people,^29^ family planning patients who identified as non-straight more commonly experienced SRH access delays.

This finding mirrors results found at the national level early in the pandemic.^2^ These findings on the link between sexual orientation and increased SRH-related delays due to COVID-19 build on existing evidence, highlighting the increased barriers and delays that sexual minorities experience accessing this care for non-COVID-related reasons, including not wanting to bother a health care provider, concerns over cost/insurance, negative prior health care experiences, and being unable to get an appointment.^30^ Taken together, all this evidence further supports bolstering efforts to expand and support access, especially for individuals who have historically experienced the greatest barriers to this access.

It is probable that uncertainty and fear regarding COVID-19 transmission played a role in individuals' delayed access to SRH care.^1^ Other likely sources of these COVID-19-related access issues seem to be better situated at the health center level, related to clinic shutdowns, staffing shortages, reduced focus on SRH care, and pivots to pandemic-related care,^4–6^ and thus solutions to addressing these delays should target these health center-level challenges to reduce impact for patients. During the COVID-19 pandemic and the ensuing delays in accessing SRH care and contraceptive methods, this study documents health care providers offering these services worked to make care more accessible, including offering more virtual and telehealth options for connecting with a health care provider and getting a method.^5,6,9,10^

Without these simultaneous efforts on the part of providers to overcome COVID-19-induced obstacles to in-person care, delays in accessing contraceptive care may have been far greater among our respondents. In addition, some types of providers—especially those who already had the infrastructure in place to pivot to telehealth contraceptive care delivery—were better able to nimbly integrate these broader service options into their offerings during the pandemic, and these provider-level differences would trickle down to patients, creating further inequities in access for individuals depending on where they seek care.^31(p19)–33^ Efforts are needed to support increased access to SRH care in innovative ways and through a diversity of options, with special attention to the needs of individuals with fewer resources.

Our study has a few important limitations to note. Our data come from a sample of individuals who had originally been recruited at health care sites that provided publicly supported family planning services, with over half of respondents in Arizona and Iowa having gotten care specifically at a Title X site. Given the Title X program's focus on ensuring access to family planning care, especially among low-income individuals, our findings do not represent experiences of the broader population of reproductive-aged individuals in these states, including those who may have been experiencing challenges to accessing SRH care before the pandemic.

Some of our findings regarding significant relationships between respondent demographics and our outcomes of interest should be interpreted with caution, given the instability in estimates reflected in somewhat broad confidence intervals. Our survey item asking about delays to SRH care due to COVID-19 did not explicitly include or exclude telehealth care, so respondents may have differentially interpreted telehealth as being included in their response. Our survey item asking about job-related reductions or losses due to COVID-19 asked about personal and close family/friend experience all together, and it is important to note that a friend's experience with this would likely have a very different impact on the individual's own financial situation than a close family or personal experience.

In addition, given the different timelines of data collection across the states, several variables of interest in our study (sexual orientation, health insurance, and employment) among Wisconsin respondents were drawn from baseline data rather than a more recent data wave as in Arizona and Iowa; given the time-variant nature of all these measures,^19,20^ findings from Wisconsin should be interpreted through a cautionary lens. Finally, because of the data collection timeline in Wisconsin, a large portion of respondents in this state may not have answered a COVID-19 item until their first monthly survey opportunity in Spring 2021 when state-wide vaccination efforts were in progress and cases were plummeting. This may lead to underestimates of the impact of COVID-19 on Wisconsinites in our study.

## Conclusions

Our findings on state-specific impact of the COVID-19 pandemic on access to SRH care over a year into the pandemic underscore the importance of one's geographic location to being able to realize full reproductive autonomy. In all three states, delays in accessing this care were more common than among the one-third of cisgender women at the national level reporting this outcome early in the pandemic.^2^

As the COVID-19 pandemic continues to evolve, with simultaneous increases in the COVID-19 Delta variant and vaccination rates and differential approaches across the country with regard to opening back up, attention to mitigating challenges experienced due to the pandemic, while anticipating potential further challenges and disruptions in SRH care, will be key. Especially, given the disproportionate impact of the pandemic on the employment and economic situation of women,^34^ LGBTQ people, and individuals experiencing financial instability, ensuring access to broad SRH care is crucial to supporting peoples' reproductive autonomy.

Importantly, our findings highlight only a small piece of the larger picture of how individuals' reproductive autonomy was impeded due to the pandemic. Further research regarding the extent to which these COVID-19-related delays resulted in subsequent negative consequences for individuals—such as having to rely on less preferred methods of contraception, forego contraception all together, and/or experience unwanted pregnancies—is warranted.

## Notes

^*^
Family planning care included the following: a method of contraception, counseling or medical testing regarding contraception, STI tests or treatment, human papillomavirus vaccination, a pregnancy test, or regular gynecological examinations. Prenatal and abortion care were not included, as patients receiving these services had a confirmed pregnancy.

^†^
Across the samples in each of the three states, we collapsed the following race/ethnicities indicated by respondents into an “other race/ethnicity” category for analytic purposes: Asian or Asian American, Native American/Alaskan Native, Native Hawaiian/Pacific Islander, mixed, mestizo, and other. This group represented less than 1% of the sample population in each of the states.

^‡^
Financial instability data represent experiences over the past 6 months for Iowa and Arizona respondents and experiences over the past month for Wisconsin respondents.
